# Cervical spine motion during tracheal intubation with videolaryngoscopy versus Macintosh laryngoscopy in adults undergoing cervical spine surgery: a randomized controlled trial

**DOI:** 10.1016/j.bjane.2026.844847

**Published:** 2026-10-15

**Authors:** Hieu Trung Doan, Thang Toan Nguyen, Anh Thi Hong Nguyen, Du Gia Hoang

**Affiliations:** a Bach Mai Hospital, Center for Anesthesia and Surgical Intensive Care, Hanoi, Vietnam; b Hanoi Medical University, Department of Anesthesia and Critical Care, Hanoi, Vietnam; c Bach Mai Hospital, Department of Orthopedic and Spine Surgery, Hanoi, Vietnam

**Keywords:** Airway management, Cervical vertebrae, Fluoroscopy, Randomized controlled trial, Tracheal intubation, Videolaryngoscopes

## Abstract

**Background:**

Excessive cervical spine motion during tracheal intubation may increase neurological risk in individuals with cervical spine pathology. Videolaryngoscopy may reduce cervical motion compared with Macintosh direct laryngoscopy, but any biomechanical reduction should be distinguished from neurological protection.

**Methods:**

Adults scheduled for elective cervical spine surgery were randomized 1:1 to intubation with a ClearVue videolaryngoscope (group V) or a Macintosh laryngoscope (group M) under manual in-line stabilization. Lateral fluoroscopy was acquired immediately before laryngoscopy (G0, neutral position) and at best glottic view (G1). The prespecified primary endpoint was directly measured cumulative C0‒C5 cervical motion. Cervical spine motion (ΔG) was also calculated at C0‒C1, C1‒C2, C2‒C3, C3‒C4 and C4‒C5. ΔG was expressed in degrees (°).

**Results:**

Fifty-six patients were randomized and analyzed (28 per group). Mean (SD) ΔG at C0‒C5 was 19.71 (4.75) in group V and 26.32 (8.46) in group M (mean difference [M‒V], 6.61; 95% CI 2.91‒10.31; p < 0.001). At C0‒C1, ΔG was 2.68 (2.53) vs. 4.68 (2.42) (difference = 2.00; 95% CI 0.67‒3.33; p = 0.004). First-attempt success was 89.3% (25/28) vs. 78.6% (22/28) (p = 0.47). Among patients intubated successfully on the first attempt, intubation time was shorter in group V (21.75 ± 3.64 s) than group M (31.71 ± 21.25 s).

**Conclusion:**

ClearVue videolaryngoscopy reduced fluoroscopically measured upper cervical and directly measured cumulative C0‒C5 motion compared with Macintosh direct laryngoscopy. These findings should be interpreted as biomechanical surrogate outcomes, not as evidence of neurological protection.

## Introduction

Tracheal intubation in individuals with cervical spine pathology is challenging because laryngoscopy can produce cervical spine extension and translation, particularly at the upper cervical segments.^1^, ^2^, ^3^ Although the motion threshold associated with neurological deterioration is unknown, minimizing cervical spine motion is widely advocated in both traumatic and non-traumatic cervical spine disease.^1^ In a cadaver model with a destabilized cervical segment, common airway maneuvers produced measurable motion across the injured level, supporting the rationale for motion-minimizing techniques.^4^ Awake flexible bronchoscopic intubation is often recommended when feasible; however, direct laryngoscopy under manual in-line stabilization (MILS) remains common in many operating room and emergency settings.^1^

Videolaryngoscopes may provide an improved glottic view with less lifting force and potentially less upper cervical motion compared with Macintosh direct laryngoscopy, although results vary by device design and immobilization strategy.^5^, ^6^, ^7^, ^8^, ^9^, ^10^, ^11^ Evidence specific to individual videolaryngoscope designs remains clinically relevant because blade geometry, optical axis, tube-shaping strategy, and operator familiarity can influence both glottic exposure and cervical spine kinematics. We designed this randomized trial to compare cervical spine motion measured by lateral fluoroscopy and intubation performance using a ClearVue videolaryngoscope versus a Macintosh laryngoscope in adults undergoing elective cervical spine surgery with MILS.

We hypothesized that videolaryngoscopy would reduce directly measured cumulative cervical spine motion (ΔG at C0‒C5) during laryngoscopy compared with Macintosh direct laryngoscopy.

## Methods

### Study design, ethics, and registration

This prospective, single-center, parallel-group randomized controlled trial was conducted at Bach Mai Hospital. Patient recruitment and enrollment occurred between February 2024 and September 2024. The study protocol had been approved before patient enrollment by the Ethics Committee at Bach Mai Hospital (Decision n° 4665/QĐ-BM, dated August 14, 2023; project code: BM_2023_23). All patients provided written informed consent. The trial was conducted in accordance with the Declaration of Helsinki.

The ethics-approved protocol prespecified the directly measured cumulative C0‒C5 cervical spine motion as the primary outcome, the segmental cervical spine motion and intubation performance outcomes as secondary outcomes, and included the sample-size assumptions and statistical analysis plan before enrollment began. No changes were made to the trial design, outcomes, sample-size assumptions, or statistical analysis plan after patient enrollment started. Recruitment ended when the planned sample size was achieved.

The trial was not prospectively registered in a public registry. Retrospective registration was completed in the Australian New Zealand Clinical Trials Registry (ANZCTR; ACTRN12626000389303; registered on March 27, 2026). The full protocol is available from the corresponding author upon reasonable request.

### Patients

Adults aged 18 years or older scheduled for elective cervical spine surgery under general anesthesia were screened. Inclusion criteria were ASA physical status I‒II and consent to participate. Exclusion criteria were rapid-sequence induction requirement, intubation without neuromuscular blockade, limited mouth opening (< 2 cm), body mass index > 35 kg.m^-2^, history of cervical spine or maxillofacial surgery, ankylosing spondylitis, or acute cervical spine trauma with established spinal cord injury.

Cervical pathology was classified preoperatively as traumatic cervical spine injury or non-traumatic pathology based on the surgical diagnosis. Non-traumatic indications included cervical disc herniation, cervical spinal stenosis, and cervical spinal cord tumor.

### Randomization and blinding

Patients were randomized 1:1 to videolaryngoscopy (group V) or Macintosh direct laryngoscopy (group M) using a computer-generated block randomization table created before recruitment by an investigator not involved in patient enrollment. Allocation assignments were placed in sequentially numbered, opaque, sealed envelopes, which were opened immediately before induction after eligibility confirmation and consent. Because the devices differ, the intubating anesthesiologist could not be blinded.

To minimize performance variability, all tracheal intubations were performed by the same study anesthesiologist, who had more than 5 years of anesthesia and intensive care experience and was familiar with both Macintosh direct laryngoscopy and the ClearVue videolaryngoscope. The fluoroscopy operator and the assessor who measured cervical angles were blinded to group assignment. The intubator was not allowed to view fluoroscopy images during intubation.

### Anesthesia, airway devices, and MILS protocol

Standard monitoring included electrocardiography, pulse oximetry, and noninvasive arterial blood pressure. Neuromuscular transmission was monitored with TOFscan (Dräger). Patients were positioned supine on a firm horizontal table with head and neck in neutral position. After preoxygenation (6 L.min^-1^ for 3‒5 minutes), anesthesia was induced with propofol 2 mg.kg^-1^ and fentanyl 3 µg.kg^-1^, followed by rocuronium 0.6 mg.kg^-1^. Mask ventilation was performed after apnea; an oral airway was permitted if needed. Laryngoscopy began after confirmation of complete neuromuscular blockade (TOF count 0).

Tracheal intubation was performed using either a ClearVue videolaryngoscope (Infinium Medical, USA) or a Macintosh laryngoscope according to allocation. The ClearVue device used a non-channeled, hyperangulated D-blade (size 3 or 4) with a distal camera near the blade tip. In group V, a malleable stylet was inserted into the tracheal tube and precurved to approximately 60 degrees to match the hyperangulated blade curvature and optical axis. In group M, a standard Macintosh blade was used, and the tracheal tube was shaped with a malleable stylet into a hockey-stick configuration with an approximately 35-degree distal bend. These device-specific features were considered when interpreting generalizability to other videolaryngoscopes, particularly Macintosh-style or differently angulated devices.

Two dedicated anesthesia assistants from the spine operating room team were trained before study implementation and were responsible for applying MILS. During laryngoscopy, the assistant stood at the head of the patient beside the intubating anesthesiologist, held the mastoid processes bilaterally with the fingertips, and supported the occiput with the palms without applying traction. The same standardized MILS technique was maintained throughout induction and laryngoscopy. External laryngeal manipulation was permitted when the glottic opening was < 50% visible, but this maneuver was not systematically recorded as a separate outcome.

Intubation was limited to 120 seconds per attempt. If oxygen saturation decreased to < 90% or the time limit was reached, mask ventilation was resumed for 1-minute before reattempting intubation. If intubation failed, the assigned device was abandoned, and an alternative rescue technique was used according to local difficult-airway practice. Successful tracheal intubation was confirmed by capnography and bilateral chest auscultation.

### Fluoroscopy acquisition and cervical angle measurement

Lateral fluoroscopic images of the skull base and cervical spine were obtained at two time points: immediately before laryngoscopy with the head-neck in neutral position (G0), and at the point of best glottic view (G1). Fluoroscopy adhered to ALARA principles (tight collimation, shortest feasible exposure, and standard shielding) to minimize any incremental radiation. After confirming alignment, the operating table and fluoroscopy equipment were not moved during image acquisition.

Bone reference lines were defined using two anatomic landmarks per structure (Fig. 1), following previously described fluoroscopy-based techniques.^3^^,^^6^^,^^8^^,^^11^ Reference lines were drawn for C0 (McGregor line, between the posterior margin of the hard palate and the opisthion), for C1 (a line through the anterior and posterior arches of the atlas), and for C2‒C5 (tangential lines through the anterior and posterior endplates of each vertebral body). For each image, the absolute angle between each reference line and a horizontal baseline was measured.

**Figure 1:**
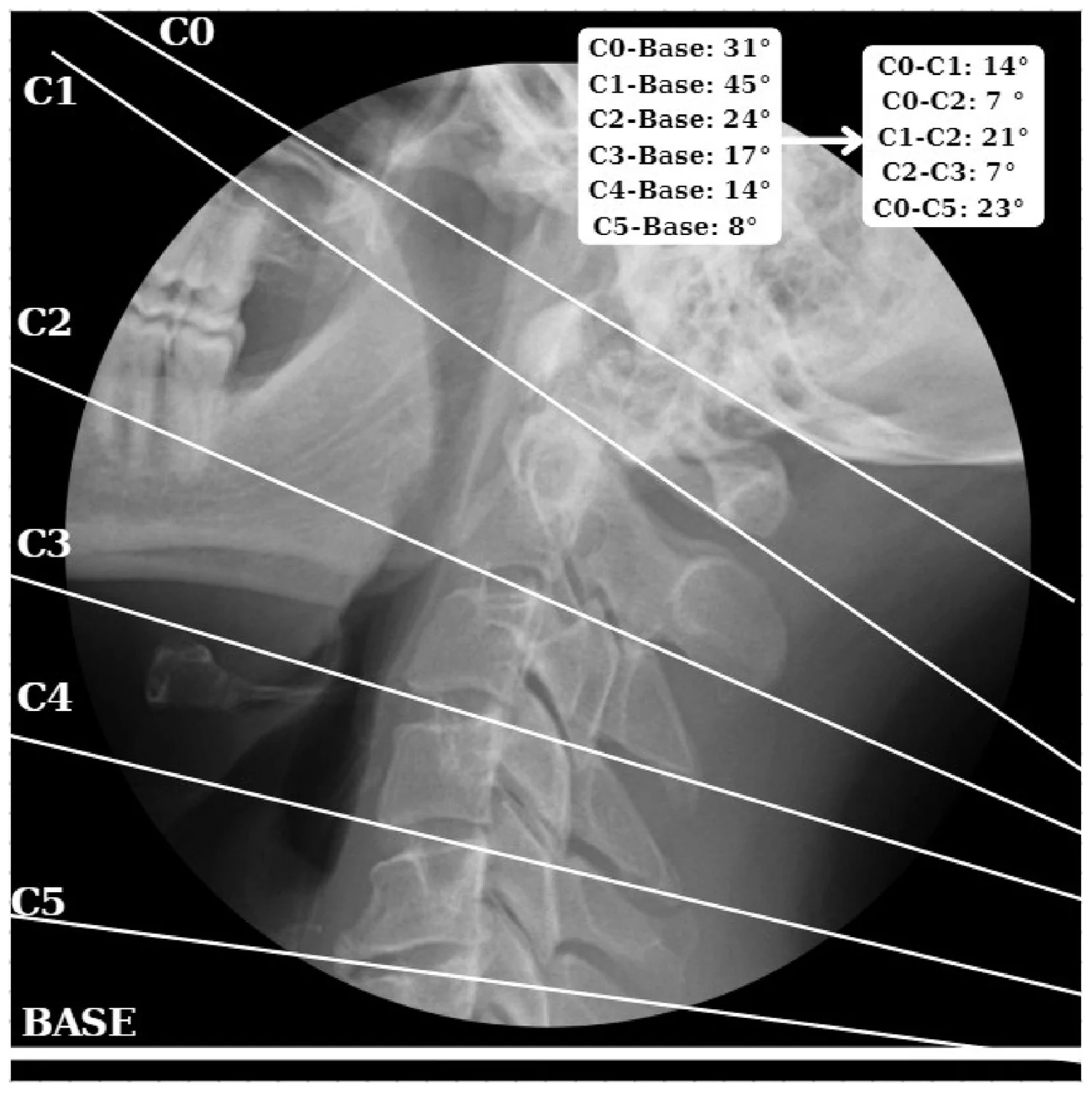
Reference lines and angle measurements on lateral fluoroscopy used to calculate intervertebral and directly measured cumulative cervical motion (ΔG).

Intervertebral segmental angles (C0‒C1, C1‒C2, C2‒C3, C3‒C4, and C4‒C5) were calculated from adjacent reference lines. Segmental motion was reported as the absolute angular change between G0 and G1 for each segment. The prespecified cumulative C0‒C5 outcome was calculated directly at the patient level as the absolute change in the global C0‒C5 angle between G0 and G1, using the C0 and C5 reference lines. Therefore, the C0‒C5 value was not derived by summing group-level mean segmental values; the latter can differ from the direct composite measurement because adjacent segments may move in different directions and because absolute segmental magnitudes do not preserve directionality.

All measurements were performed by a blinded assessor. Each angle was measured twice and averaged to reduce measurement error. Formal intra-observer reliability statistics (e.g., intraclass correlation coefficient) were not prespecified and were not calculated; this is acknowledged as a limitation.

### Outcomes

The prespecified primary outcome was directly measured cumulative cervical spine motion (ΔG) at C0‒C5. Secondary outcomes included segmental ΔG at C0‒C1, C1‒C2, C2‒C3, C3‒C4, and C4‒C5; first-attempt intubation success; intubation time; glottic view assessed using the Cormack-Lehane grade; and perioperative airway-related adverse events.

First-attempt success was defined as successful tracheal tube placement on the first laryngoscopy attempt, confirmed by continuous capnography. An intubation attempt was defined as insertion of the laryngoscope into the mouth; removal of the device and/or resumption of mask ventilation marked the end of an attempt. Intubation time was measured in seconds from mouth opening/laryngoscope insertion to confirmation of successful tracheal intubation by capnography. Glottic view was recorded at the point of best glottic view. Prespecified safety outcomes included oxygen desaturation < 90%, dental injury, mucosal bleeding, and perioperative airway-related complications.

### Sample size

Sample size was informed by prior cinefluoroscopic studies comparing airway devices,^3^^,^^6^ and by the sample-size approach used in recent cervical motion trials.^11^ Although the prespecified primary endpoint was the directly measured cumulative C0‒C5 motion, the planning assumptions were based on C0‒C1 motion because the occipito-atlanto-axial complex is clinically important, highly mobile during laryngoscopy, and better characterized in prior radiographic studies than composite C0‒C5 motion. A between-group C0‒C1 difference of 2.0 degrees was used for sample-size planning based on prior radiographic studies, with an assumed SD of 2.5 degrees. With a two-sided alpha = 0.05 and 80% power, 25 patients per group were required. We planned to recruit 28 patients per group (total 56), allowing for approximately 10% attrition or missing fluoroscopy images. The primary C0‒C5 result is interpreted using the observed mean difference and 95% confidence interval rather than post-hoc power.

### Statistical analysis

Analyses were performed using SPSS (version 20.0) and were conducted on an intention-to-treat basis. Continuous variables are presented as mean ± SD and categorical variables as counts (%). Between-group comparisons of continuous outcomes used Welch’s *t*-test (or Mann-Whitney *U*-test if non-normal). Categorical outcomes were compared using Fisher’s exact test. Effect sizes are reported as mean differences with 95% confidence intervals for continuous outcomes and odds ratios with 95% confidence intervals for categorical outcomes. A two-sided p-value < 0.05 was considered statistically significant.

Given multiple cervical segments were evaluated, the directly measured cumulative C0‒C5 motion was treated as the prespecified primary endpoint. Segmental analyses (C0‒C1, C1‒C2, C2‒C3, C3‒C4, and C4‒C5) were secondary, exploratory and interpreted accordingly. No formal adjustment for multiple comparisons was applied. The statistical tables and SPSS output were independently checked by a second author.

## Results

Between February 2024 and September 2024, 62 patients were assessed for eligibility. Six were excluded (ankylosing spondylitis, n = 2; acute cervical spine trauma with established spinal cord injury, n = 2; obesity, n = 1; declined participation, n = 1). Fifty-six patients were randomized, allocated, and analyzed (28 per group). No patients were lost to follow-up or excluded from the primary analysis (Fig. 2). Recruitment ended when the target sample size was achieved.

**Figure 2:**
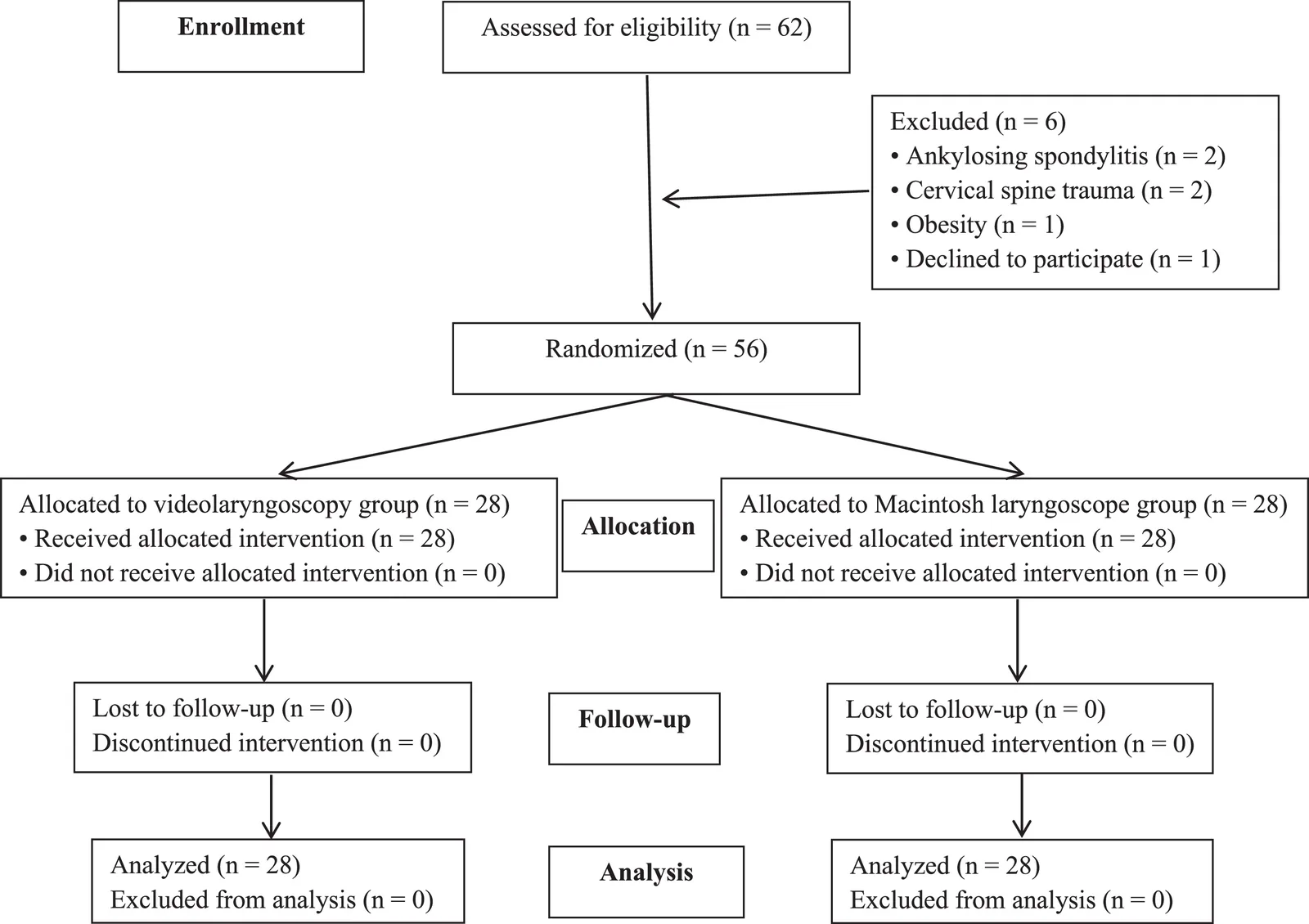
CONSORT flow diagram of patient recruitment, allocation, follow-up, and analysis.

Baseline demographic and airway characteristics were similar between groups (Table 1). Traumatic cervical pathology was present in 9 patients (32.1%) in each group. Non-traumatic pathology was present in 19 patients (67.9%) in each group and included cervical disc herniation, cervical spinal stenosis, and cervical spinal cord tumor.

**Table 1:** Baseline characteristics and cervical pathology.

Variable	Videolaryngoscope (V) (n = 28)	Macintosh (M) (n = 28)
Age, years	53.32 ± 15.67 (22‒78)	54.89 ± 13.23 (22‒75)
Male sex, n (%)	17 (60.7)	18 (64.3)
Weight, kg	53.89 ± 7.98 (40‒70)	55.96 ± 8.27 (36‒71)
Height, cm	158.00 ± 9.81 (145‒177)	157.03 ± 8.91 (143‒175)
BMI, kg.m⁻²	21.74 ± 3.60 (16.7‒30.1)	23.75 ± 4.65 (16.3‒33.2)
ASA I/II, n	10/18	9/19
Traumatic cervical pathology, n (%)	9 (32.1)	9 (32.1)
Non-traumatic cervical pathology, n (%)	19 (67.9)	19 (67.9)
Mouth opening, cm	3.66 ± 0.52 (3.0‒4.5)	3.71 ± 0.51 (3.0‒4.5)
Thyromental distance, cm	6.58 ± 0.85 (5.0‒8.0)	6.55 ± 0.80 (5.0‒8.0)
Mallampati I/II/III/IV, n	4/17/5/2	5/18/4/1

Non-traumatic cervical pathology included cervical disc herniation, cervical spinal stenosis, and cervical spinal cord tumor. Sex, ASA physical status, and Mallampati class are reported as categorical distributions. Continuous variables are presented as mean ± SD (min-max) and categorical variables as n (%).

### Cervical spine motion

Videolaryngoscopy was associated with lower directly measured cumulative C0‒C5 motion than Macintosh direct laryngoscopy (Table 2). Mean difference (M‒V) at C0‒C5 was 6.61 (95% CI 2.91‒10.31; p < 0.001). At C0‒C1, the mean difference was 2.00 (95% CI 0.67‒3.33; p = 0.004). Segmental differences at C1‒C2, C2‒C3, C3‒C4, and C4‒C5 were not statistically significant; C4‒C5 motion was 3.21 ± 2.45 in group V and 3.66 ± 2.96 in group M (mean difference [M‒V], 0.45; 95% CI -1.00 to 1.91; p = 0.54) (Table 2).

**Table 2:** Cervical spine motion during tracheal intubation.

Segment	ΔG, V (mean ± SD) °	ΔG, M (mean ± SD) °	Mean difference (M−V), ° (95% CI)	p-value
C0‒C1	2.68 ± 2.53	4.68 ± 2.42	2.00 (0.67‒3.33)	0.004
C1‒C2	15.86 ± 7.37	16.75 ± 7.89	0.89 (-3.20 to 4.98)	0.66
C2‒C3	2.14 ± 2.49	2.18 ± 2.52	0.04 (-1.30 to 1.38)	0.95
C3‒C4	1.43 ± 1.71	1.68 ± 1.94	0.25 (-0.73 to 1.23)	0.61
C4‒C5	3.21 ± 2.45	3.66 ± 2.96	0.45 (-1.00 to 1.91)	0.54
C0‒C5	19.71 ± 4.75	26.32 ± 8.46	6.61 (2.91-10.31)	< 0.001

Delta G (ΔG) denotes the change in intervertebral angle between the neutral position immediately before laryngoscopy (G0) and the point of best glottic view (G1) on lateral fluoroscopy. Positive values indicate extension. Between-group comparisons were performed using Welch's *t*-test. The C0‒C5 outcome was measured directly at the patient level from the C0 and C5 reference lines and was not calculated by summing group-level mean segmental values.

C1‒C2 showed the largest segmental motion in both groups. This pattern was verified during data review and is consistent with the biomechanical predominance of the upper cervical spine during laryngoscopy, especially around the occipito-atlanto-axial complex, and with prior fluoroscopic studies showing that airway instrumentation concentrates motion in upper cervical segments.^3^^,^^4^^,^^6^^,^^8^^,^^13^

### Intubation performance and safety

First-attempt success and glottic view favored videolaryngoscopy numerically but did not reach statistical significance (Table 3). Among patients intubated successfully on the first attempt, intubation time was shorter in group V than group M (21.75 ± 3.64 seconds [range 17‒29] vs. 31.71 ± 21.25 seconds [range 14‒106]; mean difference [M‒V], 9.96 seconds; 95% CI 0.45‒19.47; p = 0.041). No rescue airway technique, protocol deviation, or study-related adverse event was observed (including desaturation < 90%, dental injury, or mucosal bleeding).

**Table 3:** Intubation performance and safety.

Outcome	V (n = 28)	M (n = 28)	Effect estimate	p-value
First-attempt intubation success	25/28 (89.3%)	22/28 (78.6%)	OR = 2.27 (95% CI 0.51‒10.18); RD 10.7%	0.47
Cormack-Lehane grade III–IV	4/28 (14.3%)	9/28 (32.1%)	OR = 0.35 (95% CI 0.09‒1.32)	0.21
Intubation time among first-attempt successes, seconds (s)	21.75 ± 3.64 (17‒29); n = 25	31.71 ± 21.25 (14‒106); n = 22	MD = 9.96s (95% CI 0.45‒19.47)	0.041
Rescue airway technique	0/28	0/28	Not estimable	‒
Protocol deviation	0/28	0/28	Not estimable	‒
Study-related adverse events	0/28	0/28	Not estimable	‒

OR, Indicates odds ratio comparing group V vs. group M; RD, Indicates risk difference (V‒M); MD, Indicates mean difference (M‒V). Fisher's exact test was used for categorical p-values; Welch's *t*-test was used for intubation time.

## Discussion

In this randomized trial in adults undergoing elective cervical spine surgery with MILS, ClearVue videolaryngoscopy reduced both directly measured cumulative cervical motion (C0‒C5) and upper cervical motion (C0‒C1) at the time of best glottic view compared with Macintosh direct laryngoscopy. The between-group difference in the primary outcome was 6.61 (95% CI 2.91‒10.31), and the C0‒C1 difference was 2.00 (95% CI 0.67‒3.33). These results support the biomechanical premise that videolaryngoscopy can reduce cervical extension required to obtain an adequate glottic view.

The finding should be interpreted in the context of prior fluoroscopic and radiographic studies, in which indirect or optical laryngoscopic devices generally reduced upper cervical extension or composite cervical motion compared with Macintosh laryngoscopy under immobilization or MILS.^6^^,^^8^^,^^10^^,^^12^^,^^13^, ^14^, ^15^ The present study adds device-specific evidence for the ClearVue hyperangulated videolaryngoscope, but its clinical novelty is incremental rather than definitive; similar biomechanical patterns have been reported with other videolaryngoscopes and optical devices. Differences among studies likely reflect device geometry, blade curvature, tube-shaping strategy, immobilization method, and operator experience.

The C1‒C2 values were larger than adjacent lower cervical segmental values in both groups. This distribution is plausible because the occipito-atlanto-axial complex is the most mobile part of the cervical spine during airway instrumentation, and because laryngoscopic force directed at the tongue base and vallecula can be transmitted primarily to upper cervical segments.^3^^,^^4^^,^^6^^,^^8^^,^^13^ The addition of C2‒C3, C3‒C4, C4‒C5 data confirms that lower cervical motion was smaller and did not differ significantly between groups, whereas the largest inter-device difference was concentrated at C0‒C1 and the directly measured composite C0‒C5 outcome. Our findings are consistent with prior fluoroscopic and radiographic studies comparing videolaryngoscopes or optical laryngoscopes with Macintosh laryngoscopy under immobilization or MILS, which generally report reduced upper cervical extension and/or reduced composite cervical motion with indirect laryngoscopy.^6^^,^^8^^,^^10^^,^^12^ In contrast, flexible bronchoscopy techniques may minimize cervical motion even further, but can be less practical in time-critical scenarios and require specific expertise and preparation.^1^^,^^5^^,^^13^ A plausible mechanism for reduced motion with videolaryngoscopy is the lower lifting force required to obtain an adequate glottic view, because less force may translate into less cervical extension and less bending moment at the cervical spine.^7^ This biomechanical relationship has been demonstrated experimentally and provides a physiologic rationale for preferential use of videolaryngoscopy when minimizing cervical motion is prioritized.

Contemporary difficult-airway guidance increasingly incorporates videolaryngoscopy into structured airway strategies rather than treating it as a rescue-only device.^16^^,^^17^ Recent expert guidelines on universal implementation of videolaryngoscopy similarly emphasize equipment standardization, training, availability, and team-level implementation.^18^ Therefore, the present results should not be interpreted as a device-only solution. The potential benefit of videolaryngoscopy in cervical spine pathology depends on standardized MILS, adequate neuromuscular blockade, gentle technique, operator expertise, ergonomics, team communication, and early rescue planning.^19^

Although first-attempt success and Cormack-Lehane grade favored videolaryngoscopy numerically, the trial was not powered for intubation performance outcomes. The shorter first-attempt intubation time observed with videolaryngoscopy should also be interpreted cautiously because the analysis included only patients successfully intubated on the first attempt. Nonetheless, the direction of effect is consistent with evidence that videolaryngoscopy can improve glottic visualization in immobilized or anticipated difficult airway conditions.^9^^,^^12^

Most importantly, reduced angular cervical motion is a biomechanical surrogate outcome and should not be interpreted as evidence of neurological protection. The threshold of cervical motion associated with neurological deterioration remains unknown, and this study was not powered to detect neurological complications. Device choice should therefore be integrated into a broader movement-minimizing airway strategy rather than used as the sole determinant of safety.

### Limitations

This study has limitations. First, the intubator could not be blinded to the device, introducing potential performance bias, although all intubations were performed by the same experienced anesthesiologist using a standardized protocol. Second, cervical spine motion was derived from two static fluoroscopy time points (neutral position and best glottic view) rather than continuous imaging; therefore, peak motion during tube advancement may not have been captured. Third, formal intra-observer reliability statistics were not prespecified, although each angle was measured twice by a blinded assessor and averaged. Fourth, the trial was not prospectively registered, which may raise concerns about selective reporting despite prior ethics approval, prespecified outcomes, and subsequent retrospective registration in ANZCTR. Fifth, the study enrolled ASA I‒II adults undergoing elective cervical spine surgery and excluded severe obesity, ankylosing spondylitis, acute cervical spine trauma with established spinal cord injury, and anticipated highly difficult airway situations; therefore, findings may not be generalizable to emergency airway management, acute unstable cervical trauma, established spinal cord injury, severe obesity, high ASA physical status, or physiologically unstable patients. Finally, we did not directly measure laryngoscopic lifting force, hemodynamic responses, sore throat, hoarseness in a structured manner, radiation dose, or patient-centered neurological outcomes.

## Conclusion

In adults undergoing elective cervical spine surgery with MILS, tracheal intubation using the ClearVue videolaryngoscope was associated with reduced fluoroscopically measured upper cervical (C0‒C1) and directly measured cumulative C0‒C5 motion compared with Macintosh direct laryngoscopy. These findings represent biomechanical surrogate outcomes and should not be interpreted as proof of neurological protection. Future studies should evaluate continuous motion profiles, broader airway populations, and patient-centered neurological outcomes.

## Data availability statement

The de-identified numerical data underlying the reported tables and the statistical output files are available from the corresponding author upon reasonable request after publication, subject to institutional policies, approval of a scientifically sound proposal, and completion of an appropriate data-sharing agreement. Because the dataset comes from a single-center clinical trial with a relatively small sample size, unrestricted public deposition was not undertaken at this stage to minimize the risk of indirect participant identification. The authors will comply with any additional data-sharing requirements requested by the journal.

## Trial registration

Australian New Zealand Clinical Trials Registry (ANZCTR): ACTRN12626000389303; registered on March 27, 2026. The trial was retrospectively registered.

## Ethics approval

Bach Mai Hospital Ethics Committee approval (Decision n° 4665/QĐ-BM, dated August 14, 2023; project code: BM_2023_23). Written informed consent was obtained from all participants.

## Use of AI and AI-assisted technologies

During the preparation of this revised work, the authors used ChatGPT (OpenAI) solely to improve language clarity, readability, and organization of the response to reviewers. The tool was not used to generate data, perform statistical analyses, or create or alter figures or results. After using this tool, the authors reviewed and edited the content as needed and take full responsibility for the integrity of the work.

## Authors’ contributions

All authors contributed to the study conception and design. Data collection was performed by the study team. Statistical analysis was performed by an author with responsibility for the analysis plan. The first draft of the manuscript was written by the authors, and all authors critically revised the manuscript and approved the final version.

## Conflicts of interest

The authors declare no conflicts of interest.
